# Bullous pemphigoid in patients who are frail: a case complicated by cerebral nocardiosis and *Pneumocystis jirovecii* pneumonia

**DOI:** 10.1093/skinhd/vzag038

**Published:** 2026-04-28

**Authors:** Simone Landini, Alessandro Magnatta, Alberto Corrà, Elena Biancamaria Mariotti, Valentina Ruffo di Calabria, Luca Sanna, Virginia Corti, Walter Volpi, Lavinia Quintarelli, Raffella Santi, Gioia Di Stefano, Marzia Caproni, Alice Verdelli

**Affiliations:** Section of Dermatology and Venereology, Department of Health Sciences, University of Florence, Florence, Italy; Section of Dermatology and Venereology, Department of Health Sciences, University of Florence, Florence, Italy; Immunopathology and Rare Skin Diseases Unit, Department of Health Sciences, Azienda USL Toscana Centro (ERN-SKIN), University of Florence, Florence,Italy; Immunopathology and Rare Skin Diseases Unit, Department of Health Sciences, Azienda USL Toscana Centro (ERN-SKIN), University of Florence, Florence,Italy; Section of Dermatology and Venereology, Department of Health Sciences, University of Florence, Florence, Italy; Section of Dermatology and Venereology, Department of Health Sciences, University of Florence, Florence, Italy; Section of Dermatology and Venereology, Department of Health Sciences, University of Florence, Florence, Italy; Immunopathology and Rare Skin Diseases Unit, Department of Health Sciences, Azienda USL Toscana Centro (ERN-SKIN), University of Florence, Florence,Italy; Immunopathology and Rare Skin Diseases Unit, Department of Health Sciences, Azienda USL Toscana Centro (ERN-SKIN), University of Florence, Florence,Italy; Section of Anatomic Pathology, Department of Health Sciences, Azienda USL Toscana Centro, University of Florence, Florence, Italy; Section of Anatomic Pathology, Department of Health Sciences, Azienda USL Toscana Centro, University of Florence, Florence, Italy; Immunopathology and Rare Skin Diseases Unit, Department of Health Sciences, Azienda USL Toscana Centro (ERN-SKIN), University of Florence, Florence,Italy; Immunopathology and Rare Skin Diseases Unit, Department of Health Sciences, Azienda USL Toscana Centro (ERN-SKIN), University of Florence, Florence,Italy

## Abstract

Bullous pemphigoid (BP) is the most common autoimmune blistering disorder in older patients and is typically managed with long-term immunosuppressive therapy tailored to the patient’s comorbidities and overall health. Patients who are frail with concurrent haematological malignancies represent a particularly vulnerable population due to their impaired immunity and treatment-related immunosuppression. We report the case of an 85-year-old man with mantle cell lymphoma, previously treated with rituximab and ibrutinib, who developed BP confirmed by histology, direct immunofluorescence, indirect immunofluorescence and enzyme-linked immunosorbent assay. Initial disease control was achieved with systemic corticosteroids and methotrexate. Subsequently, he developed a subcutaneous thoracic nodule consistent with panniculitis, followed by cerebral nocardiosis and *Pneumocystis jirovecii* pneumonia. Despite successful remission of BP, his clinical course was complicated by life-threatening infections requiring prolonged hospitalization. This case highlights the delicate balance between controlling autoimmune disease and preventing severe opportunistic infections in older, frail patients with haematological malignancies. Current BP guidelines should be refined by integrating better frailty assessment, infection prophylaxis and tailored immunosuppressive strategies.

## Teaser

A patient who was frail with mantle cell lymphoma developed *Nocardia* and *Pneumocystis jirovecii* infections following the onset and treatment of a concurrent bullous pemphigoid, highlighting the delicate balance between autoimmune control and infection risk.

What is already known about this topic?

- Bullous pemphigoid (BP) commonly affects older and frail individuals, often requiring prolonged immunosuppressive treatment.
- An immunocompromised state increases the risk of opportunistic infections, but guidelines rarely address infection prevention in patients with BP who are frail.

What does this study add?

- This case illustrates how standard immunosuppressive regimens for BP can trigger life-threatening infections in patients who are frail and have haematological malignancies.
- We emphasize the need for tailored therapeutic approaches and possible infection prophylaxis in high-risk populations.

Bullous pemphigoid (BP) is the most common autoimmune blistering skin disorder seen in the older population. It is characterized by subepidermal blisters and significant morbidity due to its chronic course and the need for prolonged immunosuppressive therapy.^1^ The pathophysiology involves the formation of autoantibodies against hemidesmosomal proteins BP180 (type XVII collagen) and BP230. The association between BP and haematological malignancies has been well documented.^2^ Mantle cell lymphoma (MCL), an aggressive B-cell lymphoma, has emerged as a particular concern owing to its frequent occurrence in older patients and the impact of immunosuppressive therapy on disease management.^3^ The use of targeted therapies such as Bruton tyrosine kinase inhibitors (ibrutinib) in MCL adds further complexity to the management of potentially associated autoimmune skin diseases.^4^ Current management guidelines for BP focus on standard immunosuppressive approaches, including systemic corticosteroids and steroid-sparing agents.^5^ However, these recommendations do not address the unique challenges faced by patients who are immunocompromised, particularly those with underlying haematological malignancies treated with modern, targeted therapies. This knowledge gap is particularly relevant as the ageing population grows, with an increasing number of older patients presenting with autoimmune diseases and haematological malignancies. Recent epidemiological studies have estimated the annual incidence of BP to range between 6 and 43 cases per 1 million inhabitants, with a marked increase among individuals aged >80 years.^6^ Furthermore, up to 15–20% of patients with BP have coexisting neurological or autoimmune disorders, and approximately 7–10% present with an associated malignancy, most frequently a haematological malignancy.^7^ These data highlight the significant clinical overlap between BP and systemic diseases in older patients who are frail, emphasizing the need for individualized management strategies.

We present a complex case that illustrates the intricate relationship between BP, MCL and immunosurveillance failure in an older patient receiving multimodal therapy. This case demonstrates the evolution of BP under immunosuppressive pressure and highlights opportunistic infections as a critical complication in this population.

## Case report

**Figure 1:**
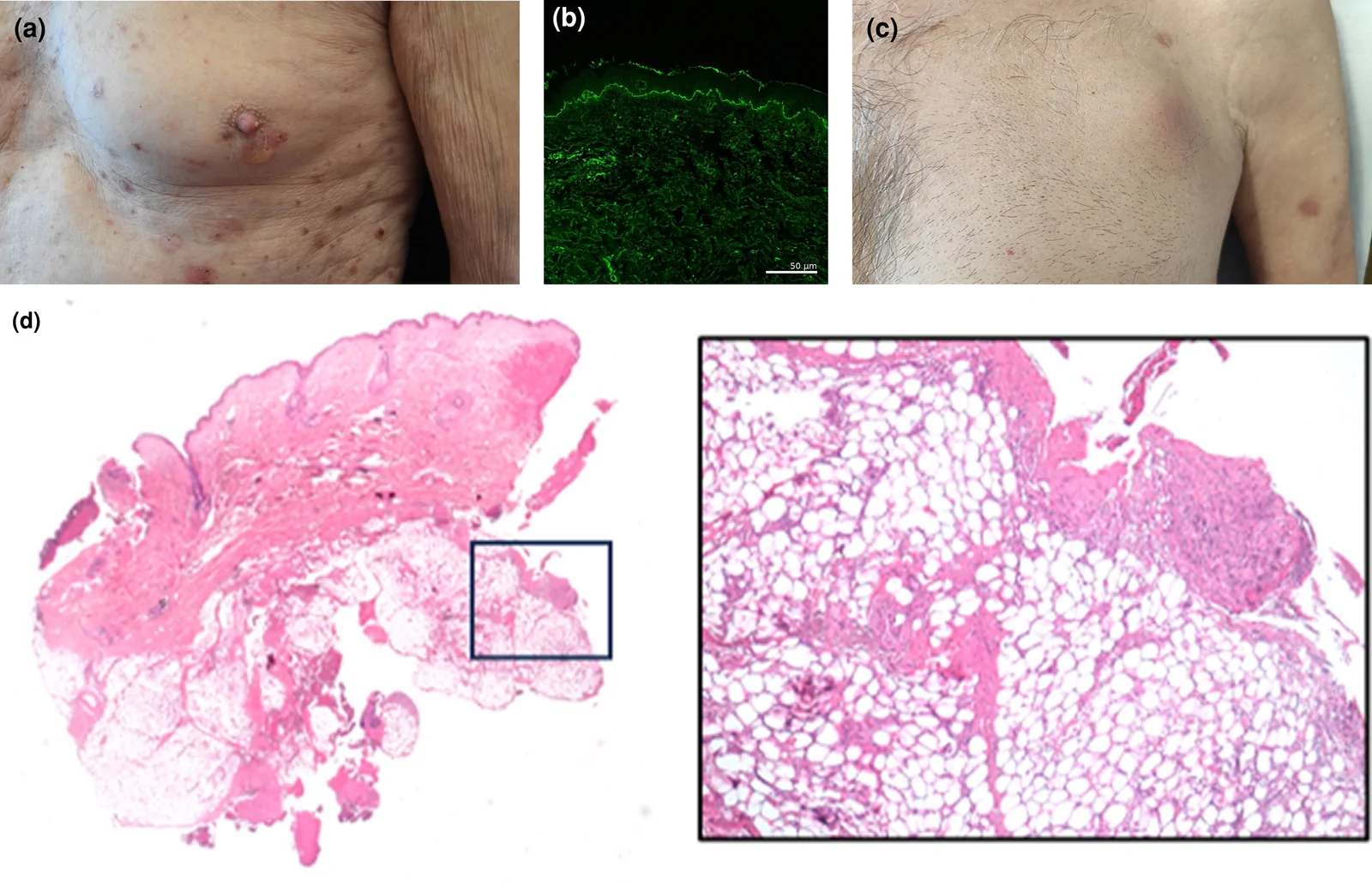
Clinical, immunopathological and histopathological findings. (a) Pruritic, tense, fluid-filled bullae on erythematous skin, predominantly involving the trunk and limbs. (b) Direct immunofluorescence showing linear IgG deposits along the epidermal side of the dermoepidermal junction; fluorescein isothiocyanate conjugated antihuman IgG. Scale bar = 50 µm. (c) Well-defined, firm nodular lesion located in the left chest area, initially suspected to represent a neoplastic lymph node due to the patient’s history of mantle cell lymphoma. (d) Skin and subcutaneous tissue biopsy (haematoxylin and eosin; low-power view, objective ×4) showing preserved epidermis, fibrotic thickening of the subcutaneous septa and an inflammatory infiltrate rich in polymorphonuclear cells, histiocytes and occasional multinucleated giant cells, with areas of liponecrosis (inset, higher magnification; objective ×20).

An 85-year-old man with a 5-year history of MCL, previously treated with rituximab and more recently with ibrutinib for disease relapse, presented to our dermatology clinic with painful erosive oral and genital mucosal lesions, followed by the appearance of pruritic, tense, fluid-filled bullae on erythematous skin surfaces predominantly involving the trunk and limbs (Figure 1a). He denied any recent change to his medication regimen.

Clinical examination revealed multiple well-circumscribed erosions and tense bullae ranging from 1 to 4 cm in diameter, symmetrically distributed over the trunk and anterior extremities. Nikolsky sign was negative, consistent with a subepidermal blistering disorder. The presence of erosive oral and genital lesions, along with patient-reported pain, suggested mucosal involvement.

A skin biopsy from the edge of a newly formed bulla on the right flank confirmed subepidermal blistering. Direct immunofluorescence showed linear IgG deposits along the epidermal side of the dermoepidermal junction (Figure 1b). Indirect immunofluorescence on salt-split skin demonstrated linear IgG deposition on the epidermal (roof) side and an enzyme-linked immunosorbent assay revealed elevated anti-BP180 IgG (92 U mL^–1^) with negative anti-BP230 IgG (7 U mL^–1^), confirming BP. At diagnosis, the Bullous Pemphigoid Disease Area Index (BPDAI) was consistent with moderate-to-severe disease [Activity (BPDAI-A) = 58, Damage (BPDAI-D) = 7].

The patient started systemic corticosteroids [prednisone 0.5 mg kg^−1^ daily (40 mg daily)]. Within 4 weeks, the lesions improved and no new bullae had formed. Prednisone was gradually tapered over 8 weeks, achieving complete resolution of cutaneous manifestations. Follow-up was initially scheduled for every 4 weeks and later extended to every 6 weeks as the patient entered remission. At this stage, his BPDAI score indicated complete clinical remission (BPDAI-A = 0, BPDAI-D = 8).

One year later, the patient experienced a relapse in his BP, which was refractory to systemic and topical steroids. After multidisciplinary consultation with haematology colleagues, methotrexate was initiated at 7.5 mg weekly as a steroid-sparing agent, resulting in complete cutaneous resolution.

Three months after the last remission, the patient noted a rapidly enlarging subcutaneous nodule in the left thoracic region (approximately 4 cm in diameter). It was firm, nontender and fixed to underlying structures (Figure 1c). The lesion gradually enlarged over 2 weeks. The initial impression was potential lymphomatous relapse due to his history of MCL. A comprehensive evaluation included surgical excision. Histopathology showed adipose tissue with inflammatory infiltrates consistent with septal panniculitis (Figure 1d). Special stains (periodic acid–Schiff, Grocott–Gömöri methenamine silver, Ziehl–Neelsen) and cultures for acid-fast bacilli and fungi were negative. Polymerase chain reaction (PCR) for *Mycobacterium tuberculosis* complex and fungal PCR on tissue were also negative, making cutaneous tuberculosis and deep fungal infection unlikely. Additional microbiological investigations did not support alternative infectious diagnoses. Immunohistochemistry showed no malignant lymphoid cells. Despite the absence of systemic symptoms, immunosuppressive therapy was discontinued and ibrutinib temporarily suspended given the patient’s stable MCL.

Subsequently, the patient experienced a syncopal episode with altered mental status. Brain imaging revealed a right temporal lobe abscess. Surgical drainage and microbiological analysis identified *Nocardia* species (*N. asteroides* complex). Intravenous trimethoprim–sulfamethoxazole was started. During hospitalization, pulmonary infiltrates developed; empirical meropenem initially improved his status, but worsening hypoxaemia prompted bronchoalveolar lavage (BAL). *Pneumocystis jirovecii* pneumonia (PCP) was confirmed by PCR of the BAL fluid. The patient required prolonged intensive care with multiple antimicrobial drugs. While he achieved complete remission of BP, the clinical course remained complicated by recurrent opportunistic infections.

## Discussion

BP is an autoimmune blistering disease that affects older, often frail, individuals or those who are in immunosenescence.^6^ Immunosuppressive therapies, especially systemic corticosteroids and methotrexate, increase the risk of opportunistic infections.^8^ The risk is amplified in patients with haematological ­malignancies like MCL, which impair immune surveillance and are often treated with biologics or targeted inhibitors.^4^

The patient’s background of immunosuppression included prior exposure to rituximab and ibrutinib, as well as recent use of systemic corticosteroids and methotrexate. While rituximab and ibrutinib may contribute to immune dysfunction,^9,10^ the development of opportunistic infections probably reflected a multifactorial susceptibility related to age, underlying MCL and cumulative immunosuppression required to control BP.

Methotrexate was chosen as a steroid-sparing agent due to its reported efficacy and predictable risk profile in patients who are frail.^11^ The achievement of complete remission supported the appropriateness of this therapeutic approach.

The subcutaneous nodular lesion, initially suspected to represent neoplastic relapse, was identified as septal panniculitis, highlighting the importance of a comprehensive differential diagnosis that includes infectious aetiologies. In retrospect, the lesion may have represented an early cutaneous manifestation preceding disseminated nocardiosis, although no pathogen was identified in the excised tissue. Nocardiosis, although rare, poses a life-threatening risk in patients who are immunocompromised, with central nervous system involvement reported in up to 44% of disseminated cases.^12^ Prompt imaging and microbiological confirmation are essential for diagnosis. Similarly, PCP may present with insidious symptoms but may rapidly progress to respiratory failure if not promptly recognized and treated.^13^

This case emphasizes the need for a multidisciplinary approach. Dermatologists, geriatricians, haematologists, infectious disease specialists and internists should coordinate therapy, monitor for early signs of infection and consider prophylaxis. Frailty assessments, such as the Clinical Frailty Scale or Frailty Index, help identify high-risk patients and guide immunosuppressant use.^14^ Current dermatological guidelines may not fully address the specific challenges faced by frail and older patients, indicating the need for updated recommendations that incorporate risk stratification and preventive strategies.

This case underscores the importance of individualized, multidisciplinary care and highlights the need for research on strategies that balance autoimmune disease management with infection prevention in patients who are frail. Current BP guidelines do not specifically address frail populations or recommend routine antibiotic prophylaxis. Future updates to BP guidelines should emphasize tailored therapeutic approaches, including the use of second-line therapies (such as omalizumab or dupilumab),^15^ as well as targeted preventive measures and the assessment of frailty.

### Author contributions

Simone Landini (Conceptualization [lead], Supervision [lead], Visualization [lead], Writing—original draft [lead], Writing—review & editing [lead]), Alessandro Magnatta (Writing—­original draft [supporting], Writing—review & editing [supporting]), Alberto Corrà (Writing—original draft [supporting], Writing—review & editing [supporting]), Elena Biancamaria Mariotti (Writing—original draft [supporting], Writing—review & editing [­supporting]), Valentina Ruffo di Calabria (Writing—original draft [supporting], Writing—review & editing [supporting]), Luca Sanna (Writing—original draft [supporting], Writing—review & editing [supporting]), Virginia Corti (Writing—original draft [supporting], Writing—review & editing [supporting]), Walter Volpi (Writing—original draft [supporting], Writing—review & editing [supporting]), Lavinia Quintarelli (Writing—original draft [supporting], Writing—review & editing [supporting]), Raffaella Santi (Writing—original draft [supporting], Writing—review & editing [supporting]), Gioia Di Stefano (Writing—original draft [supporting], Writing—review & editing [supporting]), Marzia Caproni (Conceptualization [equal], Supervision [equal], Writing—original draft [equal], Writing—review & editing [equal]), and Alice Verdelli (Conceptualization [equal], Supervision [equal], Writing—original draft [equal], Writing—review & editing [equal])

## Data Availability

The data supporting this case report are available from the corresponding author upon reasonable request.
